# Editorial: The Sensing Brain: The Role of Sensation in Rehabilitation and Training

**DOI:** 10.3389/fnins.2020.645319

**Published:** 2021-01-25

**Authors:** Susan Hillier, Geert Verheyden, Jane E. Sullivan, Leeanne M. Carey

**Affiliations:** ^1^University of South Australia, Adelaide, SA, Australia; ^2^Department of Rehabilitation Sciences, KU Leuven, Leuven, Belgium; ^3^Feinberg School of Medicine, Northwestern University, Chicago, IL, United States; ^4^Department of Occupational Therapy, Social Work and Social Policy, School of Allied Health, Human Services and Sport, College of Science, Health and Engineering, La Trobe University, Melbourne, VIC, Australia; ^5^Neurorehabilitation and Recovery, The Florey Institute of Neuroscience and Mental Health, Heidelberg, VIC, Australia

**Keywords:** sensation, perception, sensorimotor, rehabilitation, multi-modal

In the skill acquisition and rehabilitation literature substantial attention is placed on the central and peripheral action systems—the efferent and motor performance side of the behavior. However, there is a quiet but steady interest being expressed in the role of sensation—and the perception of sensation—in improving performance. There is also growing recognition that the primary function of different sensory systems can be trained in clinical populations to improve detection, discrimination, and spatial/object recognition. Moreover, the more hidden function of perception of sensations can improve motor performance by improving feedback and feedforward.

We are delighted that the following selection of 12 papers explores these topics conceptually and empirically in humans. They include a systematic review, randomized controlled trials, pilot reports, imaging, and mechanistic studies; in clinical and non-clinical populations.

Several authors have investigated aspects of sensory function in healthy populations. Zerr et al. have explored multi-sensory processing—in particular the temporal binding window—whereby we build up composite and coherent perception. Their pilot randomized controlled trial (RCT) demonstrates this window of time for binding multiple sensory input is both modifiable with training AND can influence function—in this example fluency of speech. Further, Matsugi et al. elaborate on the role of the cerebellum in modulating specifically vestibulo-spinal function using either transcranial magnetic (repetitive cerebellar) or galvanic (noisy) stimulation. Both authors invite readers to consider future clinical applications.

Yasuda et al. and Zhang et al. both considered the sensory aspects of walking in healthy populations. Yasuda investigated augmenting feedback (vibrotactile) to the feet during gait practice whilst Zhang tested a system that simulates the usual tactile pressures experienced on the soles of the feet during walking. Lopez-Rosado et al. explored these ideas in a pilot clinical trial with people with stroke—finding that increasing tactile input to the affected foot (using a sock that passively stimulates) could improve gait speed.

Other papers also investigated the role of sensory training in people with stroke. To provide an overview of the current literature (Serrada et al.) have reported a systematic review and meta-analyses of RCTs investigating the effect of sensory-based interventions on function after stroke. They confirmed that the literature still supports the effectiveness of more passive modalities—stimulating body parts with afferent (sub-motor-level) devices—with the strongest evidence. There is also emerging evidence for the active retraining of sensory appreciation and its effect on both sensory appreciation and motor performance, however more research in larger, stronger studies is still needed.

Other studies investigated specific modalities for sensory-based intervention post-stroke. Gandolfi et al. concluded that robotic stair walking (as an example of assisted, task-specific training) improved both postural control and sensory integration in their RCT. Allowing the participants to have a voice in their RCT, Turville et al. reported that people post-stroke found their somato-sensory retraining program (SENSe) to be both challenging and rewarding—believing it helped them to increase their sensation and to improve the way they used their stroke-affected arm. Adding to the quest for robust methodology in sensory training research, Carey et al. validated an objective measure of haptic object identification for researchers and clinicians to use to evaluate change in stroke survivors before and after the same form of somatosensory retraining.

The final stroke-related paper investigated a different form of somato-sensory appreciation. Cai et al. concluded that people with stroke can consistently and accurately perceive force production (torque or effort) at either their affected or less affected elbow if tested unilaterally, but not simultaneously. This confirms we need to further consider the role of cognition and attention in sensory rehabilitation.

The remaining paper leads us back to considering multiple sensory input. Cuppone et al. investigated the idea that people with visual impairments may also have spatial perceptual issues. They used an intact sense (auditory) coupled with movement to improve proprioceptive spatial perception in this group. This furthers our understanding of the way we can use complementary senses to substitute in situations of severe impairment.

We hope you find these studies of interest—we are particularly pleased that the research spans different sensory modalities from vision and audition to somatosensory and vestibular. We look forward to ongoing interest in the training of sensation (and perception) for improvement of skilled performance in non-clinical and clinical populations.

## Author Contributions

All authors contributed to the editorial and approved the final version.

## Conflict of Interest

The authors declare that the research was conducted in the absence of any commercial or financial relationships that could be construed as a potential conflict of interest.

